# American Medical Association

**Published:** 1860-08

**Authors:** 


### SELECTIONS.

### American Medical Association.

*Thirteenth Annual Meeting, held at New Haven, June 5th, 6th and 7th,* 1860.

SECOND DAY.

WEDNESDAY, June 6th, 1860.

The Association was called to order at the Chapel of Yale College, at 9 o'clock A. M., by Vice-President Dr. Wilson Jewell, of Pa. The minutes of the previous day's proceedings were read and adopted.

On motion of Dr. Gardner, of Mass., the Rules of Order were suspended, to allow V. P. Dr. Logan to offer a resolution. Dr. Logan then tendered his resignation as Vice-President, stating that while he appreciated the honor, he found himself surrounded by circumstances which had determined him not to retain the office. In justice to himself, in addition, he felt it necessary to state *emphatically, that it was a position he had neither sought or desired.* The resignation was accepted.

The Committee on Education, through Dr. Reese, of N. Y., Chairman, made their report.

The report urged the adoption of a higher standard of qualifications than are now considered requisite. The present winter sessions were considered too short, and they thought the session should continue six or nine months, and there be fewer lectures on each day.

They urged the introduction of chairs on various subjects, now not taught in medical schools, such as hygiene, medical literature, &c.

The report considered sufficient attention was not paid to clinical (at the bedside) instruction, and that the required curriculum of study should be extended to various collateral branches, without a knowledge of which, no man could properly perform his duties as a physician; even though he possess diplomas from regular medical schools; and they urged upon the Association the importance of taking prompt and immediate action to increase the standard of the profession, as whatever reform was to be instituted, should emanate from this body, and the matter should be takon in hand immediately, instead of appointing successive committees, thus incurring a year's delay, again and again.

The Committee desired to be considered as having no desire for any fastidions reform, but only desired such changes as would increase the general interests of the profassion.

In conclusion, the Committee offered the following preamble and resolutions as part of their report:

Preamble.—*Whereas*, It is the deliberate judgment of the American Medical Association, that the time has fairly come for the introduction of improvements into the present system of medical education, which shall elevate the existing standard of qualification for the Doctorate, and especially for securing and encouraging a higher degree of attainment in the science and of skill in the art of medicine than has been heretofore accessible to students in our country, and

*Whereas*, This body of American Physicians is regarded by our fraternity everywhere, as the acknowledged head and representative of the medical profession in the United States, and it is therefore looked to for prescribing the terms and qualifications of those who are henceforth to be admitted and recognized into our fellowship as brethren and equals in the profession; therefore,

*Resolved*, That it be hereafter regarded as an indispensible pre-requisite to enrolment as a student of medicine in the office of any regular physician, that the party shall be at least seventeen years of age, of good moral character and habits, and shall have received a good English, classical and mathematical education, and be able to read and translate the Latin language, and have an elementary knowledge of Greek, so far, at least, as to be able to trace the derivations from it to the English language.

*Resolved*, That this same requisite be made indispensable before matriculation in any regular medical college can be allowed, and that the faculty of such college, and the preceptor of such candidate for enrolment, be required to ascertain such qualification by actual examination, and to certify thereto.

*Resolved*, That the term of study in the office of a regular practitioner, including attendance upon lectures, be, and is hereby extended to four years, the last year to be mainly employed in receiving clinical instruction in medicine, surgery, and midwifery.

*Resolved*, That three full courses of lectures, in a regularly incorporated college, or other body of lecturers recognized by the Association, be required of all candidates for the Degree of Doctor of Medicine. Said candidate may be admitted to examination after three full years of study, on all the branches which they have been required to study, except clinical medicine, as above.

*Resolved*, That the period of instruction in every college be extended through the full term of nine months in each year, and that this time be divided into two sessions, the first to be chiefly occupied in instruction in the elementary branches only, and the latter to the practical and more complete branches. Those in attendance upon the former to constitute the junior class, and that upon the latter, the senior. Not more than four lectures to be delivered upon each day in either of the departments, and that each lecture be preceded by a recapitulation, in the form of question and answer of the lectures of the day before.

*Resolved*, That the number of professors in each college should be increased, so as to bear some proportion to the largely increased number of branches, a knowledge of which is necessary. This increase to be made in addition to those holding clinical chairs.

*Resolved*, That the examination of all the students for matriculation, which admits them into the junior class, shall be repeated before their entrance into the senior class, either by the faculty, or by examiners appointed by them for the purpose, who shall certify in the one case to the fullness of their preliminary education, and in the other to their improvement, under courses of instruction in the junior or elementary examination. Similar examinations should be required at the commencement of each session, as to the improvement made in the preceding term.

*Resolved*, That the final examination for graduation, if made by the faculty, should be in the presence of each other, and should be witnessed and certified by a board or committee of equal numbers, to be appointed for the purpose by each State Society, within whose bounds any college may be located, or by the faculty, and without whose approval the degree should not be conferred. Due notice to be given by the faculty of the time and place for the examination, and each candidate to be separately examined.

*Resolved*, That no medical college be recognized by the American Medical Associatiation to be complete in its organization, and prepared to furnish the requisite instruction, which does not either possess a hospital of its own or which has not made arrangements with a hospital contaihing not less than 80 beds, for the students of the college receiving regular clinical instruction, before being licensed to practice.

*Resolved*, That the so-called "College Clinics" cannot, in any useful or practical sense, be looked on as furnishing an adequate substitute for the clinical teaching required.

*Resolved*, That this Association regard with marked disapproval a practice which prevails with some of the faculties of the schools, viz; Of examining those students who are candidates for a degree, before the expiration of the regular session, and while the lectures are still in progress.

*Resolved*, That the titles of the several chairs in a school, as announced in its curriculum, ought to indicate a real teaching of the branches thus virtually promised to be taught, and not be assumed merely in conformity with former usage, or to gratify the temporary whim of a professor, to have an appendage to the title of his chair, while in the very next year he may abandon, and consent to its being appended to some other chair, or to its being omitted entirely in the next annual announcement. We may instance this, attaching physiology to anatomy, the latter being the substantive branch, and of itself taking up the whole time of the professor during the the entire session, which is still too short for its legitimate purposes. Still more common and misleading is the appendage of diseases of women and children to midwifery, and that of medical jurisprudence at one time to materia medica, at another to midwifery, at a third to chemistry.

All of which is respectfully submitted.

(Signed) D. MEREDITH REESE, Ch'n (N. Y.)

JOHN BELL, (Phil.)

W. K. BOWLING, (Tenn.)

Z. PITCHER, (Mich.)

CHAS. FISHBACK, (Indiana.)

*Committee on Medical Education.*

On motion of Dr. McDowell (Mo.) the Association went into Committee of the Whole to consider the above resolutions, and after some debate arose, reporting progress, and asked leave to sit again.

Report of Committee on Medical Literature was referred to the Committee on Publication without reading.

The Committee on Nominations reported that they recommended the next meeting of the Association to take place in Chicago. Ill., on the first Tuesday in June, 1861.

They nominated the following officers:

*Secretaries*—S. G. Hubbard, Conn., H. A. Jackson, Ill.

*Committee of Arrangements*—N. S. Davis, Ill., G. W. Freer Ill., De Laskie, Willon, Ill., E. Andrews, Ill., H. W. Jones, Ill., Thos. Bevans, Ill., J. Bloodgood, Ill.

*On Prize Essays*—Dan'l Brainard, Ill., D. L. McGuigan, Iowa, M. L. Leaton, Mo., John Evan, Ill., A. L. McArthur, Ill.

*Committee on Publication*—F. G. Smith, Pa., Caspar Wister, P., S. C. Hubbard, Conn., R. J. Breckenridge, Ky., Edward Hartshorne, P., H. F. Askew, Del.

*Vice-President*—In the place of Dr. Logan, resigned, R. D. Arnold, Ga.

These officers were elected by acclamation.

Committee on Prize Essays reported they had received no essay, in their opinion, worthy of awarding a prize, which re-report was referred to the Committee on Publications.

The Rules of Order were suspended to bring up the third resolution of Dr. Davis, of Illinois, laid on the table yesterday.

The resolution was at length adopted under the following form:

*Resolved*, That all essays, voluntary communications and reports, except those of officers of the Associations, reports of committees on medical education, medical literature and prize essays, shall be first presented to the Association and referred to the appropriate section, in which they shall be examined and discussed; after which they shall be returned to the Secretary of the Association, accompanied by an expression of opinion as to whether they are worthy of publication or not, and the Secretary shall pass all such designated to be worthy directly to the Committee on Publication; and others not so designated shall be retained by the Secretary or returned to their authors, as the latter may dictate.

Dr. Lewis A. Sayre, (N. Y.) who was appointed a Special Committee on Morbus Coxarius and Surgical Pathology of Articular Inflamation, read his report, which was confined to the first subject, giving an account of seventy-two cases, and the operations performed. Referred to the Surgical Section.

Surgical Treatment of Strictures of the Uretha—James Bryan (Penn.), reported progress, and asked for longer time. Referred to its proper section.

Drainage and Sewerage of large cities, their influence on Health—A. J. Semmes, Cornelius Boyle, G. M. Dove, D. C., reported progress and asked for longer time.

Puerperal Tetanus: its Statistics, Pathology and Treatment—D. L. McGuigan, Iowa; report the same as above.

Hospital Epidemics—R. K. Smith, Penna; laid over.

Puerperal Fever—S. N. Green, Ind., laid over.

Anæmia and Chlorosis—H. P. Ayers, Ind.; reported progress and asked to continue the committee to report next year.

Veratrum Viride—J. B. McCaw, Va.; laid over.

Alcohol; its Therapeutical Effects—J. W. Dunbar, Md.; asked for a change in the title, making it read, "Alchol in its relations to man;" granted. Report next year.

Meteorology—J. G. Westmoreland, Ga.; laid over.

Milk Sicknes—Robert Thompson, Ohio; partial report made; accepted and referred to section of Practical Medicine.

Manifestations of Disease of Nervous Centres—C. B. Chapman, Wisconsin; laid over.

Microscopic Observations on Cancer Cells—Geo. N. Norris, Alabama, chairman, asked to resign; committee discharged.

Philosophy of Practical Medicine—James Graham, Ohio; laid over.

On some of the Peculiarities of the North Pacific and their relations to Climate—William H. Doughty, Georgia; absent.

On the Microscope—John C. Dalton, Jr., N. Y., David Hutchinson, Ind., A. R. Stout, Cal., Calvin Ellis, Mass., Christopher Johnston, Md.; report next year.

Diseases and Mortality of Boarding Schools—C. P. Mattingly, Ky., Dixi Crosby, N. H.; reported progress; referred to its proper section.

On various Surgical Operations for Relief of Defective Vision—M. A. Pallen, Mo., T. J. Cogley, Ind., W. Hunt, Penn.; laid over.

On the Blood Corpuscle—W. Sager, Michigan; referred to proper section, with additional time.

American Medical Necrology—C. C. Cox, Md. Report was ordered to be read before the Convention, Thursday; amended to have Dr. Cox retained as chairman, and report next year.

Effects of the Virus of the Rattlesnake, when introduced in the System of Mammalia—A. S. Payne, Va.; reported progress and was discharged.

Constitutional Origin of Local Diseases, and the Local Origin of Constitutional Diseases—W. H. Mckee, N. C., C. F. Haywood, N. Y.; laid over.

Subcutaneous Injections as Remedials—I. Langer, Iowa; not allowed to report, not being an accepted delegate.

Quarantine—D. B. Clark, Pa., E. M. Snow, R. I., W. Jewell, Pa., E. D. Fenner, La., I. W. Houck, Md.; asked to be continued. Agreed to.

Medical Ethics—B. F. Schenck, Pa., chairman, resigned, and asked that Dr. Paul F. Eve, of Tennessee, be substituted; agreed to. Report next next year.

Tracheotomy in Membranous Croup—A. N. Dougherty, N. J. Partial report; this was accepted, and referred to the Surgical Section. Further time allowed to make out the report.

Effect of Perineal Operations for Urinary Calculi upon Procreation in the Male—J. S. White, Tenn. Letter from Dr. White read; laid over.

Mercurial Fumigation in Syphilis—D. W. Yandell, Ky.; laid over.

Cause and Increase of crime—W. C. Snead, Ky.; asked to be continued. Agreed to.

Education of Imbecile and Idiotic Children—H. P. Ayres, Ind. Report offered; referred to its proper section.

Pons Varolii—Partial report. The Committee wished to be continued; agreed to. Referred to Section on Anatomy.

The Committee on Voluntary Communications reported that they had received several communications on different subjects, which were referred to their appropriate sections.

Several reports and abstracts of reports on special subjects were presented, and referred to their appropriate sections.

One o'clock, the hour of adjournment, having arrived, a motion to continue five minutes longer prevailed. A little general business was then transacted, and the Convention adjourned.

There was no general meeting of the Association on the afternoon of the 6th inst., as by a resolution passed in the morning, the different sections to which special papers were referred, met to discuss the particular subjects allotted to their consideration, the members of the Association distributing themselves, and visiting that section in whose deliberations they felt most interest. These sections met in different rooms, as follows:

Section on Anatomy and Physiology, in President Woolsey's Lecture Room.

Section on Surgery, in the Geological Cabinet.

Section on General Medicine, in the Geological Cabinet.

Section on Materia Medica, in the Laboratory.

Section on Meteorology, Medical Topography, Epidemic Diseases, Medical Jurisprudence and Hygiene, in the Laboratory.

THIRD DAY.

The Association was called to order at 9 A. M., by the President, Dr. Eli Ives; afterwards, Dr. Jewell, of Philadelphia, presided.

The minutes of the previous day's proceedings were read by the first Secretary, Dr. S. G. Hubbard, of New Haven.

A list of newly registered delegates was read, making the number over five hundred.

On motion of Dr. Arnold, of Georgia, it was resolved that no communication read before the Association should occupy more than ten minutes in its reading, and no speaker should occupy the floor longer than ten minutes.

On motion of Dr. Shattuck, of Massachusetts, the rules of order were suspended, in order to allow Dr. Bowditch, Chairman of the Committee appointed to take into consideration the propriety of contributing in the erection of a suitable memorial to John Hunter, in Westminster Abbey, to present his report.—On motion, it was resolved that the Committee on Nomination be requested to consider the report and resolutions attached to it, and report thereupon, presenting the names of one from each State represented, who shall be empowered to take such action in the matter as may be hereafter agreed upon by the Association.

The Committee of Conference appointed to confer with the Committee of medical teachers reported through their chairman that they had several meetings in New York and New Haven, during which the subject of medical education had been fully discussed. He stated that in the convention of Teachers the following resolutions were adopted:

"1st. *Resolved*, That the Medical Colleges represented in this Convention, are willing to adopt the rule, if it be recommended by the American Medical Association, that every candidate for degree of Doctor in Medicine must present certificates of having assiduously studied medicine during the period of three full years under the direction of a regular practitioner of medicine, recognized as such by the American Medical Association, who shall certify to the same under his own hand, and of attendance on two *full* courses of medical lectures in a medical school, recognized as regularly organized by the American Medical Association, with an interval of at least three months between the termination of the first course and the commencement of the last.

2d. *Resolved*, That the medical colleges represented in this Convention, are willing to keep a register of their students, in which shall be entered the name, the age, the period of commencing medical studies, and diploma already received, with the name of the college conferring it, and the name of the preceptor.

3d. *Resolved*, That the medical colleges represented in this Convention, allowing that the proposed plan of admitting delegates from State Societies to attend the examination of the candidates for the degree of Doctor in Medicine to have been successfully carried out in several places, do not think that it can with advantage be universally adopted; but, at the same time, they are ready to ascertain and discuss any other measure by which the admission of unsuitable and unworthy members within the ranks of the profession can be prevented.

4th. *Resolved*, That this Convention earnestly recommend the American Medical Association to adopt such measures as will secure the efficient practical enforcement of the standard of preliminary education adopted at its first organization in May, 1847, or of a standard put forth by the medical society of the State in which a college is located, and, that medical colleges will thankfully receive and record the certificates alluded to in said standard, and one of moral character, whenever the profession generally, and the preceptors will see that students are properly supplied with them.

5th. *Resolved*, That Hospital Clinical instruction constitutes a necessary part of medical education, and that every candidate for the degree of Doctor in Medicine, shall be required to have attended such instruction regularly for a period of not less than four months.

6th. *Resolved*, That the members of this Convention are ready to co-operate in any efforts by which the attention of the community and of legislatures shall be called to the importance of the endowment of medical colleges and professorships.

7th. *Resolved*, That the attention of the American Medical Association be called to the proofs, in a letter from a German Medical Professor, of the degree of Doctor in Medicine being conferred in Germany on unsuitable persons to be used in this country."

[At the meeting of the Convention of Medical Teachers, during the discussion on the above resolutions, Prof. Logan, of Georgia, offered the following as a substitute:

WHEREAS, it is apparent that the medical colleges of the United States are not disposed to adopt the measures indicated by the American Medical Association, for the establishment of a *uniform* system of medical education, as manifested by the failure upon the part of a large portion (and among the number some of the most prominent) to be represented at the Convention of Colleges, held last year in Louisville, and by a renewal of the same course of action towards the adjourned meeting of said

Convention, and as no action on the part of the colleges represented would be likely to effect any change in the present system of medical education, and any attempt on the part of this limited representation to initiate any reform might be regarded as an offensive assumption of power; therefore,

*Resolved*, That this body declines to act for the medical colleges of the United States.

*Resolved*, That in the medical colleges, alone, resides the power of effecting any desirable change in the present system of medical education, and it is only from their united action that any good result can be expected.

*Resolved*, That a committee of ——— be appointed to report the action of this body to the American Medical Association.

The substitute was discussed by Profs. Logan, Shattuck, Crosby, McGugin, McDowell, Storer and Palmer, and was finally rejected.]

The chairman then read the following communication, addressed to the Convention of Teachers, by Prof. Henry N. Frost, of the Medical College of South Carolina, in regard to medical education in the South:

"I should wish to be heard while I make a few remarks on the progress of medical education at the South, and the advances we have made in fulfilling the requirements of the Association.—The report in my hand of the Dean of the Medical College of the State of South Carolina, of the graduates of that college and their requirements, presents a total of 114 graduates—all of whom had a preparatory education, such as the Association requires. Nearly all, with the exception of six, have had good literary opportunities; some graduates of colleges—others of academies of high repute—others instructed in the classics.—Even those whose studies were confined to English, have had their minds strengthened by the study of mathematics.

"In making this statement, I would not be understood to say that they were well versed in the classics; but they have enjoyed the opportunity, and profited in a greater or less degree by it. Neither would I be understood to say, that our graduates are all doctors. The diploma conferred is only an evidence that they have undergone a course of study; that they have been instructed in the principles of the profession, and made acquainted with the means by which they are to arrange and systematize the various occurrences presented to them; in short, that the foundation has only been laid by which they are to pursue advantageously their researches, and act for themselves. To be able doctors and successful practitioners, requires years of study and observation, and there are many who, after all this application, have never been made doctors.

The community in which a young graduate resides, soon becomes aware of this fact; it is only after a long apprenticeship, and years of toil and devotion to his business, that he acquires practice and confidence. Confidence is proverbially a plant of slow growth, and it is only after the individual has proved himself worthy that it is freely bestowed. Still, however, every doctor has been a student, and as such has to endure taunts and imputations as to his qualifications. I well remember when a student in medicine, forty-seven years since, fashionable ladies commented upon the homely appearance and neglected dress of the students of Philadelphia, and tauntingly remarked that there was little to be observed in the streets but dogs and Virginia doctors! Yet from these classes, of whom these remarks were made, there came forth a Wood, Mitchell, Meigs, McClelland, Hodge, Bartons, Derach, and, not to forget my own section, Dickson, Holbrook, Ramsay, and many others. Yet these young men were as ungainly as many at the present day; but they contained the gem, as many at the present day, which required only to be polished. Education has been progressive to my observation; our graduates show their desire to excel by seeking opportunities abroad for greater acquirements. In my day, our reading was desultory and without system. My preceptor pointed to his library, and told me to select my reading. My anatomical studies were pursued with a scalpel and the Dublin dissector. Our clinical instruction was nothing virtually. Mark the difference at the present time: your winter and summer courses; your crowded hospitals; your private instructions, and your model plates, &c. All these speak trumpet-tongued, that the work of improvement is onward."

In conclusion, the committee offered the following resolutions for adoption by the Association:

*Resolved*, That it is the duty of medical colleges to require of every candidate for the degree of Doctor of Medicine, certificates of study during the full period of three years, under the direction of a regular practitioner of medicine, recognized by the American Medical Association, who shall certify, under his own hand, as to an attendance on two full courses of lectures, with an interval of at least three months between the termination of the first and the commencement of the second course.

*Resolved*, That every medical college shall keep a volume, in which every medical student presenting himself, shall enter his name, his age, the period of his commencing the study of medicine, any diploma he may have received in evidence of previous education, with the name of the college or school from which he received such diploma; and the name of the preceptor with whom he has been studying.

*Resolved*, That hospital clinical instruction constitutes a necessary part of medical education, and every candidate should be required to have attended such instruction regularly for a period of not less than four months.

*Resolved*, That the professors of every medical college should recommend to their trustees, or board of managers, the adoption of a rule authorizing them to allow the attendance of two or three delegates, from the State Medical Society, at all examinations of candidates for the degree of the doctorate, and accord to these delegates a vote on the question of recommending such candidates for a degree.

*Resolved*, That every State Society be recommended to choose proper delegates at its annual meeting, to attend the examination of candidates for the degree of M. D., at all the medical colleges within their respective States.

*Resolved*, That this Association will not recognize as a regular organization, any college which does not require evidence of suitable preliminary education from all applicants for collegiate medical instruction.

*Resolved*, That we commend the use of all proper efforts, by which the attention of persons of means and liberal disposition, as well as legislative bodies, shall be directed to the propriety of endowing such medical colleges, and professorships thereof, as shall be recognized by the Association.

*Resolved*, That this Association recognize as a regularly organized medical college, one which has been represented at any meeting of this Association, and which complies with the preceding rules and directions.

*Resolved*, That this Association recognize as regular practitioners of medicine, all who have been members of this Association, and have not forfeited their rights and privileges, and all members of State and County Societies, in full standing.

The report was received, and taken up by sections. When the first resolution came up, a motion was made to amend, by striking out that part requiring an interval of three months to elapse between the termination of the first course and the commencement of the second; the objection being that the resolution, if adopted as offered, would do an injustice to summer schools, whose sessions would have to begin three months after the closure of the winter sessions, in order to graduate students, thus throwing the session into July, August and September, and crowding upon the next winter session; and that such a course would drive students altogether from the summer schools.

Dr. McDowell, of Missouri, spoke in strong terms against the amendment. He despised the plan of some professors, who teaching at a winter school in the South, immediately the winter session closes, bring their half-fledged brood to a Northern summer school, and there delivering a *second* course of lectures, foist their hastily hatched students upon the medical profession. He was entirely opposed to the practice of pushing and forcing, which was becoming so rampant.

The discussion was further participated in by Drs. Shattuck, of Boston, Austin Flint, N. Y., Brodie, of Mich., Palmer, of Mich., Nourse, of Me., Atlee, of Pa., and others.

Dr. Jno. L. Atlee, of Philadelphia, would rather increase the interval to six months. Nor did he want, as others had suggested, to leave the matter to the discretion of the professors in the different schools. His experience proved that a man never becomes a thorough and proper student of medicine until *after he has attended his first course of lectures*, for he then learns what is required, and how he should direct his studies so as to profit by them; and as he studies in the interval between the collegiate courses, the demonstrations of the previous winter reappear to him as he reads his text books, giving an interest to the study he could have sought for in vain before he had attended a course of lectures. He would rather have the course of instruction lengthened to 8 or 9 months, and have a less number of daily lectures. He wanted no student to have credit for attendance on more than one full course in one year, no matter how many regular courses he may have attended, and he moved to amend the amendment by striking out "an interval of 3 months, &c.," and inserting, "and no student shall be credited for having attended more than one full course of lectures in any one year."

Dr. Palmer, of Mich., said that many of the students he had met with, voluntarily placed an interval of more than one year between their two courses of lectures. That they only became students in truth after attending one course of lectures, and they would be seen at college one winter and be missed the next, reappearing the third winter, when they would come forward and bear off the honors of the school.

Dr. Nourse, of Me., thought the appetite for legislation was too great. He wanted no restrictions to the aspirant for medical honors. He objected to the amendment as unjust to poor students, who would be forced to lose one year waiting for a degree that might have been employed in some pecuniarily profitable manner, if they were allowed, as they often do, to study two years before attending lectures, often while working at the same time for support, and then having saved the sum necessary to pay for their tickets attending two courses of instruction, one following directly upon the other, thus complying with the requisites of three years study and attendance on two full courses of lectures, even though they attend two full courses in one year.

A motion of Dr. Bennett, of Danbury, Conn., to lay the whole matter on the table, was lost.

Dr. Johnson, of Mo., did not want the courses to follow too closely on each other. Apart from other considerations, such a course would crowd the student too much, and over-tax his powers of physical and mental endurance. Students required time for relaxation, whether they wanted it or not, and therefore he was in favor of a considerable interval between the courses, and only one course in a year.

Dr. Worthington Hooker, of New Haven, Conn., thought there was not too much actual instruction being crowded together that was to be avoided, but rather too much lecturing, which brings different subjects in too close connection before the minds of the students, and taxes their energies too much, and therefore he did not want the courses crowded on each other; nor did he want the lectures to be as crowded as they usually are. He stated that in the medical department of Yale College it was customary to make a distinction in the ability for receiving instruction, between medical students who had received the advantages of a previous classical education, and those who had not enjoyed this privilege; and that they considered classical students full one year in advance of the others, and that they made this distinction in their favor regarding the length of time required to be devoted to the special study of medicine. They only required two years' application from classical students, while they exacted three years' study from all others. He firmly believed that a difference of one year should be made, but he would prefer the course of application to be extended one year longer in each case, thus making it three and four years' study instead of two and three, as it now is.

Dr. Atlee was here allowed to alter his amendment so it should read, "and no student shall attend a second course of lectures until a year shall have elapsed since the commencement of the first course."

Dr. Davis, of Illinois, thought that the design of the resolution had been generally misunderstood by the members of the Association. He considered that the great fault in medical education, which they were trying to discover and rectify, consisted in the laxity exhibited in exacting from each student of medicine a suitable amount of preliminary education. He wanted a previous education, and he knew that if energetic young men love the profession of medicine and desire to become enrolled among its votaries, they would take care to obtain the necessary education if they did not possess it; and those who did not think the profession worthy of this trouble, were much better out of it; and if the preliminary education was exacted, we should have an intelligent and educated body of physicians, and not be obliged to accept among us those of whose general literary attainments we are ashamed.

He also disliked the plan of delivering the same course of lectures to students of the first and second course. Those who had studied medicine but six months, could by no manner of means comprehend what was intended for students of three years standing; and it was folly to demand it of them. He wished the colleges to be so re-organized, that there should be a gradation of instruction according to the advance made by the several classes of students.

Dr. Reese, of N. Y., remarked, that as the winter schools closed in March, and the summer schools could not, by the resolution, begin in June, this would force students to employ in study the months of July and August, which is the general period of relaxation from labor—and thus virtually, in a great measure, prevent graduation at the summer schools.

After some more discussion, the amendment of Dr. Atlee was adopted, when a motion was made by the opponents of the amendment, to lay the *resolution* on the table. This motion was lost, and finally, the *resolution*, *as amended*, *was adopted.*

Second resolution adopted.

Third resolution adopted.

The fourth resolution was amended by Dr. McCaw, requiring "that in those States where there are regular State Medical Societies, the delegates elected to be present at the examinations of candidates for the degree of M. D., in all the medical schools of the State, should be selected from the members of the State society; in those States where there are no societies, the selection is to be made from members of the profession in good standing."

Dr. Jno. L. Atlee wanted no representation of examiners from any except State Medical Societies, which would force those States where they did not exist, to organize State Medical Societies, and therefore he opposed the amendment, though he favored the original resolution.

Dr. Storer, of Mass., wished no restrictions to be placed on medical schools.

Dr. Mussey, of Ohio, wanted to have a resolution passed, providing for the examination of teachers before they were elected to professorships, and to pass no men but those who were known to be favorable to the ideas of the Association, regarding medical instruction.

Here a tumultuous clamor arose, during which the amendment was withdrawn, and the previous question called for and sustained; when the resolution was, upon call, again read, and finally adopted as originally reported.

The fifth resolution gave rise to a good deal of discussion as to the propriety and the right of placing medical schools under the censorship of the State Medical Societies.

Dr. Timothy Childs, of Berkshire, Mass., stated, that forty years ago he called for a board of examiners to be present at all examinations for a degree, and that he had never ceased to urge the propriety of so doing. He had never passed a student without such a supervision.

He stated that he was the first man to introduce into medical colleges a Professorship on Pathology, and he was always in favor of enhancing the dignity and worth of his profession, and as long as he was able to raise his voice, he would oppose to the utmost all those who attempt to lower the standard of medical excellence, regardless of the motives that prompt them to do so.

Dr. Worthington Hooker, of New Haven, Ct., explained that Yale College, further back than forty years ago, had, of its own accord, adopted the plan contained in the resolution under consideration, and during his connection with the college, there had not been one whisper of disapprobation regarding it. There was harmony between the State Medical Society and the institution, which feels the genial effects of that harmony, which gives it its strength and position.

He thought that all medical colleges should be closely watched by the State Medical Societies of their respective States.

The resolution was adopted.

Sixth resolution adopted.

Seventh resolution adopted.

Eighth resolution adopted.

The ninth resolution was, after some little discusssion, on motion, laid upon the table.

The whole report was then adopted and referred to the Committee on Publication, for publication in the forthcoming volume of Transactions.

The committee on nomination then reported the following appointments on Standing and Special Committees, which was received and adopted, and the nominations accepted.

*Committee on Medical Literature.*—Frank H. Hamilton, N. Y. Edward Warren, Md. Chas. A. Lee, N. Y., J. W. C. Ely, R. I., E. H. Clark, Mass.

*Committee on Medical Education.*—Levin T. Joynes, Va., Christopher C. Cox, Md., J. C. Bradbury, Me., L. H. Steiner, Md., M. A. Patten, Mo.

On the Surgical Treatment of Strictures of the Urethre—Jas. Bryan, Pa.

On Drainage and Sewerage of Large Cities—their inflence on public health—A. J. Semmes, La., C. Boyle and C. M. Dove, D. C.

On puerperal Tetanus—its statistics, pathology and treatment —D. L. McGurgin, Iowa.

On Anæmia and Chlorosis—A. P. Ayres, Ind.

On Alcohol and its Relations to Man—J. W. Dunbar, Mn.

On Milk Sickness—Robt. Thompson, Oramel Martin, Ohio, S. W. Bemis.

On Microscopic Observations on Cancer Cells—Geo. W. Norris, Pa.

On Blood Corpuscles—A. Sager, Mich.

On the Hygenic Relations of Air—C. C. Cox, Md., Chas. W. Parsons, R. I.

On Quarantine—D. D. Clark, Pa., M. Snow, R. I., Wilson Jewell, Pa., E. D. Fenner, La., J. W. Houck, Md.

On Medical Ethics—Paul F. Eve, Tenn., J. A. Murphy, Ohio, N. L. Linton, Mo., T. S. Powell, Ga., B. F. Schenck, Pa.

On Tracheotomy in Membranous Croup—A. N. Dougherty, N. J., Geo. H. Gray, Mass., J. M. Minor, N. Y.

On the Effect of Perineal Operations for Urinary Calculi upon Procreation in the Male—J. S. White, Tenn., J. B. McCaw, Va., R. C. Foster, Tenn.

On Mercurial Fumigations in Syphilis—D. W. Yandell, Ky.

On the Cause and Increase of Crime, and its mode of punishment—W. C. Sneed, Ky.

On the Microscope—R. C. Stiles, Vt.

On Gangrene of the Lungs—C. L. Allen, Vt.

On the Relations which Electricity sustains to the Courses of Disease—Isaac Capelbury, Ind.

On the Morbid and Therapeutic Effect of Verbal and Moral Influences—Alfred Hitchcock, Mass.

On the Causes of the Extinction of Aboriginal Races, more especially of the Red Men of America—Geo. Suckley, N. Y.

To report on the practical workings of the United States law relating to the inspection of drugs medicines—E. R. Squibb, New York, and F. Bowkitch, Mass., Prof. Jos. Carson, Pa.

On the Causes and Treatment of Ununited Fractures—E. K. Sanbone, and

On Diptheria—Alonzo Clark, N. Y.

On the effect of stimulants in the treatment of fractures—John W. Russel, Ohio.

On Dislocation of the hip and shoulder joints—Moses Gunn, Michigan.

To investigate the conditions demanded for a diploma of Doctor of Medicine in the various Medical Schools and Universities of Europe—J. Baxter Upham, Mass.; Robert Thompson, Ohio; George C Shattuck, Mass.

In regard to the committee on the memorial to JOHN HUNTER, the following resolutions were adopted:—

*Resolved*, That it be recommended to the different States to collect subscriptions of not more than one dollar each, from every regularly educated physician. All money so collected to be forwarded by the Chairman of the Committee hereby appointed, to the Treasurer of the HUNTER Medical Fund in London.

*Resolved*, That Drs. Henry J. Bowditch, Mass.; Amos. Nourse, Maine, George B. Twitchell, N. H., Charles Clark, Vt., G. L. Collins, R. I.; Charles Hooker, Conn.; Henry D. Bulkley, N. Y.; Wm. Elmer, N. J.; Jno. L. Atlee, Penna.; James Cowper, Del.; C. C. Cox, Md.; J. B. McCaw, Va.; Cornelius Boyle, D. C.; James H. Dickson, N. C.; H. K. Frost, S. C.; R. D. Arnold, Ga.; John Nott, Ala.; G. A. Nott, La.; W. G. Williams, Mass., C. A. Page, Mo.; J. B. Lindsley, Tenn.; R. J. Breckenridge, Ky.; J. W. Russel, Ohio; A. B. Palmer, Michigan; Calvin West, Ind.; Patrick Gregg, Ill.; D. L. McGugin, Iowa; J. B. Douseman, Wis.; D. W. Hand, Minn.; O. Harvey, Cal.; F. G. McSparack, Ark., be a committee to collect subscriptions.

A resolution was adopted to send a copy of the resolutions passed, to each Medical School in the country.

A resolution was adopted, directing that a seal of the Association be given to every Medical School in good standing, reserving the privilege of demanding the same upon sufficient evidence that the School had no longer claims to its possession.

It was moved, that in order to expedite business without a session next day, the sections meet at 2½ P. M., and at 4 P. M. the Association again convene to close business and receive their reports.

CLOSING SESSION.

The Association was called to order at 4 P. M., by V. P. WILSON JEWELL, in the chair.

Various special committees were callen upon to report, and failing to do so were discharged—other reports which had been placed on the Secretary's table were without reading, on motion, referred to the committee on Publication, with power to act.

The various sections were called upon for their reports, and the various papers respectively discussed by them were referred to the Committee on Publication.

The report of the committee on Rules of Order, lying on the table, was then called for and read, and the order of business acted upon, and the articles severally adopted, and afterwards the whole report was laid on the table.

A communication was read from the Essex Co. Medical Society, of the State of New Jersey, containing the following preamble and resolutions, for action upon by the Association:

*Whereas*—the indiscriminate sale of poisonous drugs at retail, is fraught with danger to the community; be it

*Resolved*, That in the opinion of this Association, it is the duty of the public authorites in the different States of the Union, to pass prohibitory laws against the retailing of morphia, strychnine, pressic, acid, etc., except on the written prescription of a regular practitioner of medicine, or on the personal application of a well-known citizen—and that a committee be appointed in the different States, to endeavor to carry into effect the spirit of the resolutions adopted.

The report was referred to the Committee on Publication, with power to act.

On motion of Dr. Davis, of Ill., it was decided that the committee called for, be appointed at his leisure by the President of the Association.

On motion, Dr. Cox, Md., was requested to present at the next meeting of the Association a paper on Necrology.

Dr. A. N. Dougherty, from the committee on Tracheotomy, reported from the mass of facts they had gathered with regard to the result of this operation, was 1 in 3 $\frac{4}{10}$. This statistics of cases in this country, as far as ascertained, was 16 cures out of 58 cases.

Trousseau before 1844 had 212 cases, of which there were 40 cures and 132 deaths—after 1848, he had in 49 cases 48 deaths. From 1849 to 1858, he had at the children's Hospital, at Paris, 466 cases—which resulted in 126 cures and 340 deaths. Another operator met with but 4 cures in 36 cases. Statistics of other operators were presented, and at the request of Dr. Dougherty, the report was referred back to the committee, with power to complete the report, and present the same at the next meeting of the Association.

Dr. Bell, of Brooklyn, offered the following resolution:

*Whereas*, Some of the papers submitted to this Association, require a longer period of time for their examination than the annual meetings will admit of: therefore, be it

*Resolved*, That the several sections have power to refer such papers to experts, who shall determine whether they are worthy of being referred to the Committee of Publication, for publication in the Transactions.

On motion, this was laid over to the next meeting of the Association.

A motion of Dr. Chapin, that all papers which had not been disposed of by the sections, should be referred by the committee on Publication to experts, who should report back to them, whether the papers were worthy of publication in transactions, was laid on the table.

Various rules of order and amendments to the Constitution, which had laid over from previous meetings, were again indefinitely postponed.

A communication from the Clinton County Medical Society of Iowa, to which was appended a Catalogue of the College, was read.

This communication charged the Westeın Reserve College with having exceeded its rights and privileges, in conferring the degree of the doctorate upon one Freeman Thompson, who had not come up to the requirements of their curriculum, who had not been examined by the professors in the presence of censors, and who had not been in attendance on lectures since the session of 1848–9 a single day. It stated, that at one time the Western Reserve College acknowledged the truth of the above charge, and at another time denied it.

They called the attention of the Association to this case, and desired that the Western Reserve College be refused representa. tion in the Association. Various papers were appended to the communications, substantiating the truth of the facts mentioned.

A motion was made to refer the whole subject to a select committee of three, to be appointed hereaftər by the Chairman, who should report on the same at the next annual meeting of the Association.

Dr. Davis, of Illinois, reminding the mover of the existence of a permanent Committee on Medical Ethics, created for just such purposes, the motion was altered to refer the matter to the Committee on Medical Ethics, with instructions to report at the next annual meeting, and carried.

A communication was read from the Legislature of Connecticut, stating that the Judiciary Committee had under consideration their memorial on Criminal Abortion, and asking, in order to further the matter, that a committee be appointed by the Association, to frame a bill meeting the exigencies of the case, to be presented for due consideration of the Legislature.

It was moved and carried, that the chair appoint a proper committee, to draw up such a bill as would meet the views of the Association, and present the same to the Legislature of the State.

A motion was made to alter the time of meeting from June to May, so that if the Association desire to meet in 1862, in New Orleans, they could do so before the time, when yellow fever occurs. This being an amendment to the constitution, was laid over for one year.

On motion of Dr. S. W. Butler, of Philadelphia, it was resolved, that this Association request the Convention of Medical Teachers to be perpetuated in connection with the American Medical Association, and meet in conference the day previous to the annual meetings of the Association, and report to the same.

On motion, the same committee appointed last year was continued, any vacancies occurring to be filled by the President.

On motion of Dr. J. L. Atlee, of Philadelphia, the chairman of the Committee on the Memorial to John Hunter, was empowered to fill any vacancy which may occur in that committee.

A motion by Dr. Mason, of New York, that a committee of five be appointed to prepare rules of order for the Association, and to report them at the next annual meeting, was laid on the table.

A communication from Elmira, N. Y., was read, advising the offer of a prize for the best essay on the application of mechanical contrivances in the practice of surgery, having reference to the cure or alleviation of hernia, sticture of the urethra, stone in the bladder, fractures, dislocations, &c., was referred to the Surgical Section of next year.

A vote of thanks was passed to the retiring officers, for the efficient menner in which they had performed their duties.

A resolution was passed to the effect that the thanks of the Association are due to the Faculty of Yale College, the medical profession, and citizens of New haven, for the elegant hospitality tendered to the Association; and to the proprietors of the different manufactories, for the generons manner in which they welcomed the delegation to inspect whatever of interest their factories embraced; and to the railroad and steamboat companies, who have reduced their fare on the respective routes, in favor of the delegates to this Association.

Various amendments to the constitution, laid over from last year, called up and indefinitely postponed.

Dr. Lewis A. Sayre, of New York, offered a resolution, that the Smithsonian Institute be asked to collect all the medical literature that has appeared in this country, and is scattered in various journals and periodicals, and collect it in a general library for the purposes of the profession.

On motion of Dr. Davis, of Ill., the Association went into a committee of the whole to consider the report of the Committee on Medical Education, Dr. Askew, Del., in the chair. An animated discussion ensued as to the extent of preparatory qualification, which ought to be exacted from young men designing to commence the study of medicine, but no conclusion being arrived at, the Committee rose and reported that they had considered the above report, but had no suggestions to make to the Association, and recommended the resolutions to the Committee on Publication.

Dr. Hamilton, of Brooklyn, N. Y., moved the adoption of a resolution to devise a plan for the organization of a College, or Board of Examiners, to be called the College of Physicians and Surgeons of the American Medical Association, in order to arrest all legislation which has reference to medical schools, and to determine what shall be the prerequisites to a degree of doctor in medicine. Said College to consist of one member from each State, and to meet annually, immediately before the annual meetings of the Association.

Dr. S. W. Butler, of Philadelphia, stated that the whole plan in detail, only under a different name, had been brought before the Association at a previous meeting.

Dr. Cox, of Maryland, was exceedingly surprised at the idea of such a suggestion, and spoke against it in bitter terms, though at the same time he urged the necessity of a proper preliminary education for medical students.

Dr. Thompson, of Ohio, said that the asserted prerequisites for a degree in reference to preliminary education, established, tewenty years ago, were always disregarded in his State.

After some general discussion on this subject, the Association, on motion, adjourned *sine die.*

